# Influence of the Thermoplastic Fiber Ratio on the Mechanical Properties of Recycled Carbon Fibers During the Carding Process

**DOI:** 10.3390/ma18020302

**Published:** 2025-01-10

**Authors:** Jean Ivars, Ahmad Rashed Labanieh, Damien Soulat

**Affiliations:** ULR 2461-GEMTEX-Génie et Matériaux Textiles-Gemtex, Ensait, University of Lille, F-59000 Lille, France; jean.ivars@ensait.fr (J.I.); ahmad.labanieh@ensait.fr (A.R.L.)

**Keywords:** recycled carbon fiber, carding, tensile properties, statistical distribution, Weibull

## Abstract

This study investigates the impact of carding and blending recycled carbon fibers (rCF) with crimped thermoplastic polypropylene (PP) fibers on the mechanical properties of rCF, using a Weibull statistical approach. Tensile properties of rCF were evaluated before and after carding with varying rCF/PP blend ratios (100/0%, 85/15%, 70/30%, and 50/50%). A comparison between the two-parameter and three-parameter Weibull models showed that the two-parameter model provided a better fit for rCF properties before carding. The results show that adding crimped PP fibers during carding helps to decrease the stress-at-break disparity and move their distribution to higher values. Furthermore, a slight increase in tensile modulus was observed in carded rCF, with higher PP ratios associated with smaller scatter modulus distributions. Elongation at break remained consistent, with the Weibull modulus increasing slightly with carding and the inclusion of PP fibers, indicating improved consistency. Overall, carding rCF with PP fibers helped in the mechanical property uniformity of the resulting carded webs without compromising tensile performance. This work shows the potential of the carding process with or without thermoplastic fibers to efficiently realign and give continuity to discontinuous recycled carbon fibers.

## 1. Introduction

The reuse of recycled carbon fibers (rCF) is a response to growing environmental concerns associated with the composites industry. Carbon fiber-reinforced polymers (CFRPs) are widely used in high-performance applications, such as aerospace, automotive, and renewable energy [1], due to their high strength-to-weight ratios and durability. Recycling and reusing carbon fibers represents a more sustainable alternative by reducing waste at the end of the life cycle of composite materials and decreasing dependency on virgin raw materials. The quality of the initial fiber source and the recycling method used [2] can have an impact on the variability of the tensile properties of rCF, such as breaking stress, tensile modulus, and elongation at break. The process used to realign those fibers can also have an influence on their properties.

While the potential environmental benefits of recycling carbon fibers are significant, the process also presents technical challenges. Recycling methods, such as pyrolysis [3] and solvolysis [4,5], are widely used to recover carbon fibers from end-of-life composite products or manufacturing scrap. However, these recycling processes often result in fibers that are shorter than their virgin precursors and stripped of their original surface treatments, or sizings [6], which were applied to improve fiber–matrix bonding and handling. The absence of sizing, combined with the inherent brittleness of carbon fibers, makes handling and reprocessing more difficult, particularly when recycled fibers need to be aligned or integrated into high-performance applications. Despite these challenges, strategies and techniques have been developed to reuse recycled carbon fibers in new materials: depending on the structure and the length, several processing methods can effectively reuse rCFs. For long rCFs, nonwoven technologies, for instance, involve combining multiple layers of randomly oriented fibers to create mats. This technique is simple and cost-effective, but results in limited strength and stiffness when a cross-lapping and needle-punching manufacturing route is applied [7]. Nonwoven carbon fiber structures are not only cost-effective but also versatile for a variety of applications beyond semi-structural reinforcements [8,9]. Recent advancements have demonstrated the feasibility of using hybrid nonwovens made of rCFs for electromagnetic interference shielding and sound absorption [10].

The carding process is a preparatory step in the manufacturing of these materials. Carding disentangles and cleans fibers while forming a continuous web, which serves as a base for further processing. Depending on the intended application, this process can lead to different pathways: the production of nonwoven materials, or the preparation of webs with doubling and drawing steps, which improve fiber alignment and enhance structural uniformity. Another advantage of the carding process is its ability to create hybrid structures by blending rCFs with thermoplastic fibers, making it a suitable option for producing thermoplastic composites.

Various studies have already demonstrated the feasibility of carding rCFs with thermoplastic fibers. Hengstermann et al. [11] were able to card 40 mm and 60 mm long rCFs, and highlighted the influence of fiber length at the card infeed. In another study, a core-sheath yarn was produced, also with 60 mm carded rCFs in the core, with a co-polyamide as a sheath [12]. Also, it has been shown that crimped polypropylene helped with mixing and carrying rCFs, diminishing fiber breakage through the carding process [13]. This approach aligns with previous works where polyamide and polyester were used as carrier fibers in blends with rCFs [14]. The morphological and mechanical properties of the fibers and the resulting web are influenced by both the material properties and the machine parameters during carding. For example, Manis et al. [15] were able to demonstrate that a higher number of worker/stripper pairs led to a greater degradation of 60 mm fiber length. Another study looked at the influence of the distance between the worker and the stripper on the length of the fibers leaving the carding machine [12]. A higher clearance between the worker and stripper led to lower fiber length degradation.

Statistical methods help with analyzing and interpreting the variability of the mechanical properties of individual fibers. Among these, the Weibull distribution is the most commonly used in the literature to study the tensile behavior of brittle fibers, such as carbon fiber [16,17,18,19], but also for natural fibers [20,21], as it accounts for variability in fiber strength caused by defects.

This study focuses on evaluating the effect of the carding process and blending rCFs with crimped thermoplastic fibers on the mechanical properties of rCFs. Polypropylene (PP) is incorporated in the carding process of rCFs in the present study. First, the mechanical performance of rCF is assessed before and after carding across different rCF/PP blend ratios. A Weibull statistical approach is then used to analyze the variability in tensile properties, such as stress at break, tensile modulus, and elongation at break. The comparative analysis uses a two-parameter Weibull model, which is found to fit the experimental data more effectively, particularly for rCF properties before carding when fitted with the Maximum Likelihood Estimation (MLE) technique. This study provides new insights into the mechanical behavior of rCF, a topic that remains underexplored in the literature. Few studies have specifically investigated the individual behavior of fibers after processes like carding or the influence of blending ratios with thermoplastic fibers.

## 2. Materials and Methods

The carbon fibers used in this study are Standard Modulus (SM) carbon fibers reclaimed by steam thermolysis. The fibers are unsized and cut to a length of 60 mm. rCF recovered using this technique shows a slight degradation in its initial tensile properties, about 10%, a good surface condition [22], and well-preserved diameter [23]. rCF before carding’s properties are listed in Table 1.

These values are extracted from 51 tensile tests performed using the protocol detailed in Section 2.2. at a gauge length of 25 mm. These values enabled us to classify these recycled carbon fibers as low-SM-type fibers [24]. The modulus and stress values were calculated under the assumption of a circular fiber cross-section.

### 2.1. Web Manufacturing with a Carding Machine

**Figure 1:**
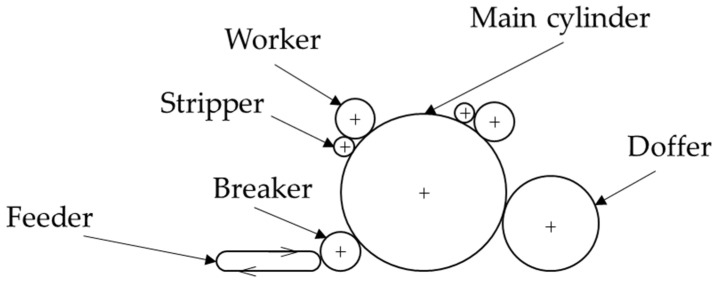
Carding machine schema.

For carding, individualize the staple fibers provided in entangled pocket, called tuft, by successive passes between toothed cylinders (Figure 1). The relative orientation of the faced cylinders permits the opening and transmission of the fibers from the feeder to the doffer and transforms the fiber in a low-inter-fiber-cohesion web. The fibers in the manufactured web are distributed randomly on the web surface. However, a preferred orientation for card manufacturing is reported for the nonwoven rCF needle punch [6].

**Figure 2:**
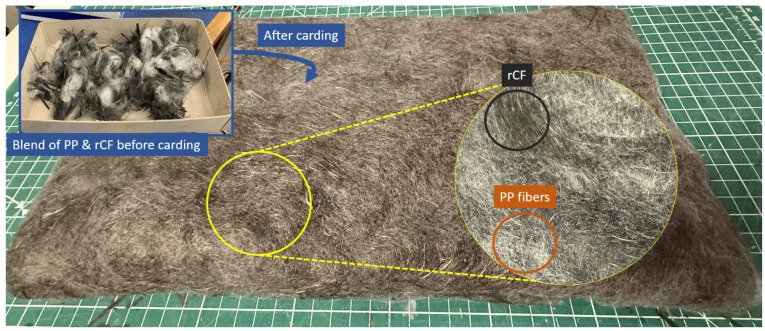
Carded web with a mix of PP and rCF.

To manufacture the web, rCF reclaimed from the steam thermolysis process are used alongside 60 mm long crimped thermoplastic polypropylene fibers at different ratios as shown in Figure 2. Polypropylene fibers are widely used in the textile industry due to its low-cost and processability, frequently used as a matrix in thermoplastic composites.

In this study, four webs are manufactured with only changing the rCF/PP blend’s ratio with, respectively, 50/50%, 70/30%, 85/15%, and finally 100/0%. Other carding parameters such as feeding density and production speed are kept constant. The physical (diameter) and mechanical tensile properties of individual fibers extracted from each manufactured web are then identified.

### 2.2. Characterization Method and Data Extraction

#### 2.2.1. Fiber Preparation and Testing

The tensile test for individual carbon fibers is performed using NF ISO 11566 standard [25], which is specific to single carbon fibers. After individualization, a single fiber is fixed to a cardboard frame using cyanoacrylate glue. The cardboard frame is mounted on an INSTRON MTS tensile tester (Norwood, MA, USA) mounted with a 10 N load cell, and the sides of the frame are cut to ensure that the load applied by the tensile tester is transmitted only to the fiber. The test speed is set at 2 mm/min and the gauge length is set to 25 mm. Cardboard frames were cut on a laser cutting machine to avoid errors in the initial length of the fibers. Doing so helps to reduce errors due to misalignment of the fiber on the cardboard frame, which can introduce measurement errors at shorter gauge lengths [26]. The compliance values were not considered for study [27,28,29].

The NF ISO 11566 standard recommends twenty measurements per fiber type. However, the inherent fragility of carbon fibers makes preparing the test difficult, potentially leading to measuring errors. Furthermore, a minimum number of tensile tests must be carried out to obtain robust data and representative results for a batch of fibers. For 50 tensile tests on carbon fiber, the Weibull modulus could vary by +/−1 [30]. Beyond 80 tests, there is no significant difference in this Weibull modulus value [18], so a balance must be struck between the time spent on the test preparation and the accuracy of the value.

Considering the number of webs and the preparation time for tensile test at fiber scale, it was considered necessary to carry out a more thorough test procedure of at least sixty tests to ensure more sufficient and reliable data.

#### 2.2.2. Diameter Measurements and Circularity Assumption

**Figure 3:**
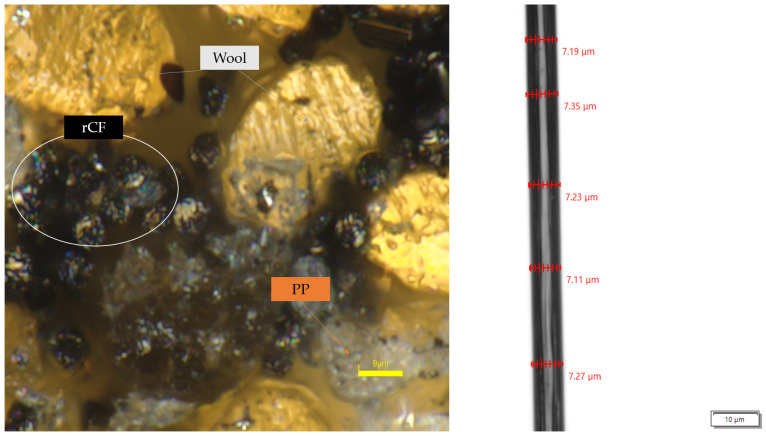
Cross-section of rCF before carding (**left**), diameter measurement of rCF before tensile testing (**right**).

For each tensile sample, diameter measurements are taken at five points along the fiber using an OLYMPUS DSX1000 microscope (Waltham, MA, USA, Figure 3, right). A circular cross-section is assumed for the carbon fibers, as it is supposed that non-circularity is not significant for the carbon fiber [26].

To support this assumption, cross-sectional measurements were performed using a microtome (Figure 3, left). The wool is added during the sample preparation step for cross-section measurements to improve the visualization of the carbon fibers. As carbon fibers are black and do not allow light to pass through, the wool provides contrast, making the fibers easier to observe. The circularity coefficient, calculated from the cross-sectional area and perimeter, reflects how close the fibers are to being perfectly circular, with a value closer to 1 indicating greater circularity [31]. This relationship is defined in Equation (1):

(1) $$Circularity=\frac{4*\pi *Area}{Perimeter^{2}}$$

Measurements of the recycled carbon fibers (rCF) before carding, carried out with ImageJ (version 1.54f), gave a circularity coefficient of 0.956 ± 0.014 across 11 samples, confirming that the fibers are highly circular prior to carding.

#### 2.2.3. Data Extraction and Analysis

Post-processing on the collected raw data, including apparent diameter, load and displacement, is performed using a Python script. The start of fiber loading is determined by identifying the second inflection point on stress–elongation curve. This eliminates the foot of the curve and ensures that all curves have the same starting point for comparison. The fiber gauge length is corrected with identified slack distance on the recorded displacement. The stress and elongation at break are determined for each test and collected for each fiber batch, per the design of the experiments. The tensile modulus is calculated between 0.1% and 0.6% of elongation [25] using a regression on the linear part of the stress/strain curve in order to avoid introducing errors due to data resolution.

These data can be used to conduct a classical comparative statistical study using mean and standard deviation for each of tensile characteristics mentioned (tensile modulus, stress and elongation at break). Additionally, it enables the carrying out of Weibull statistical modeling, suitable for the brittle behavior of fibers. Comparing Weibull parameters provides information about the variability of data distribution [17,20,30,32] and enables us to differentiate between the different batches of fibers from the webs produced. This information on the disparity in the fiber properties cannot be estimated based on the average values and standard deviations of the tensile characteristics. This provides a better understanding of how the crimped thermoplastic fibers can affect the tensile properties of the recycled carbon fiber during the carding process.

### 2.3. Statistical Analysis Using Weibull Distribution

#### 2.3.1. Introduction and Model

The elastic–brittle behavior of carbon fiber can be depicted by the Weibull’s statistic, which assumes that carbon fiber is an assembly of links. Tensile failure of the fiber occurs when the weakest link in the assembly breaks [16]. Although the Weibull distribution, used widely in carbon fiber mechanical analysis, is not an intrinsic characteristic of carbon fiber, it is a statistical model applied to assess the strength variability in carbon fiber composites which can facilitate the prediction of failure probabilities under varying stress conditions. The cumulative distribution function (CDF) is used to represent the probability of failure P at a given value of σ, the applied stress on the fiber following Equation (2), also known as the 3-parameter Weibull CDF:

(2) $$P\left(\sigma \right)=1-e^{-{\left(\frac{\sigma -\sigma_{u}}{\sigma_{0}}\right)}^{m}}$$

The 3-parameter Weibull model uses the following key parameters: $\sigma_{0}$—scale parameter (indicating the average lifetime under stress at which 63.2% of fiber are broken), m—shape of the distribution, or Weibull modulus (measuring the variability, the spread or scatter of fiber strength), $\sigma_{u}$—location parameter (reflecting the threshold for fiber failure initiation; below this value, the failure probability is null) and σ—applied stress. A Simpler form of the Weibull CDF is also considered to simulate the brittle fracture of fiber by setting the location parameter $\sigma_{u}$ in Equation (2) to zero, known as 2-parameter Weibull CDF. The location parameter in the 3-parameter Weibull distribution represents a threshold stress below which failures are unlikely to occur, enhancing the model’s accuracy, particularly when data exhibit an initial delay in failure observations. Studies on natural fibers [20,21] have demonstrated differences between the two- and three-parameter Weibull models in predicting strength, elongation at break, and tensile modulus, depending on the gauge length.

According to the different manufactured webs, these statistical models are compared with each other, but also with recycled carbon fiber tensile data before carding.

#### 2.3.2. Model Fitting on Experimental Data

One of the statistical methods used to fit a distribution to experimental data is Maximum Likelihood Estimation (MLE). It is the maximization of a likelihood function that quantifies the probability of the observed data under a particular statistical model. In the case of the 3-parameter Weibull distribution, MLE is used to determine the shape m, scale $\sigma_{0},$ and location $\sigma_{u}$ parameters, which are the most likely to produce the observed dataset [33]. Nketiah [34] reported the reliability of MLE in comparison with the Least Squares method. The likelihood function is expressed in Equation (3) with a set of points $x_{1},\;x_{2},\;\ldots ,\;x_{n}$.

(3) $$L\left(m,\;\sigma_{0},\;\sigma_{u};\;x_{i}\right)=\prod_{i=1}^{n}(\frac{m}{\sigma_{0}}{\left(\frac{x_{i}-\sigma_{u}}{\sigma_{0}}\right)}^{m-1}e^{-(\frac{x_{i}-\sigma_{u}}{\sigma_{0}}{)}^{m}})$$

Once each parameter in the Weibull distribution is determined with MLE, the next step involves the computation of the Hessian Matrix [34]. This square matrix contains the second derivatives of the log-likelihood function and is essential for establishing confidence intervals for the estimated parameters. By taking the inverse of the Hessian Matrix, the covariance matrix is obtained. This matrix contains the variances of the parameters along the diagonal and the covariances of the parameters off the diagonal. The size of the covariance matrix corresponds to the number of parameters.

For instance, in case of a 2-parameter Weibull model fitted on experimental data, the covariance matrix would take the following form, given in Equation (4).

(4) $$\left[\begin{array}{cc} Var\left(m\right) & Cov\left(m,\;\sigma_{0}\right)\\ Cov\left(m,\;\sigma_{0}\right) & Var\left(\sigma_{0}\right) \end{array}\right]$$

By using this 2 × 2 matrix and taking the square root of the variance of m and $\sigma_{0}$, it is possible to determine the standard errors of each parameter at a certain confidence interval and be able to explain the difference between the different Weibull moduli calculated for each batch of fibers before carding or extracted from webs manufactured by carding. This method was used to fit experimental tensile data for carbon fibers [19] and also natural fibers [21] with the Weibull model.

#### 2.3.3. Fitting Performance Assessment

To compare statistical distributions, one non-parametric statistical method is the Kolmogorov–Smirnoff (K-S) test. This test assesses the fit between a sample of data and a reference distribution by measuring the maximum distance between their respective cumulative distribution functions [19,21]. A higher relative value indicates a divergence between the distribution tested and the experimental data. On the other hand, a lower value suggests a higher degree of similarity between the data and the computed distribution. In addition, the *p*-value can be used to assess the significance of the model in relation to the data. A *p*-value greater than 0.05 indicates compatibility between the data and the proposed distribution. With these two metrics, it is possible to determine whether the Weibull distribution is a good fit for the experimental data. Additionally, to determine the statistical model best suited to the theoretical data, the Sum Square Error (SSE) can be calculated. This metric aims to achieve convergence by minimizing the sum of the squares of the differences between the observed and predicted values. The higher the value, the greater the disparity between the theoretical and actual values. The K-S test was conducted using a 2-parameter Weibull distribution model to analyze the static strength and fatigue life of T300 carbon fiber [35]. Similarly, Rao et al. applied the K-S test to evaluate the strength probability distribution of virgin carbon fibers, modeled with a two-parameter Weibull CDF [36].

The K-S test and the SSE values will be used simultaneously to determine the best model based on the data and the rCF ratios in the web.

## 3. Results

### 3.1. Weibull Model Selection

**Figure 4:**
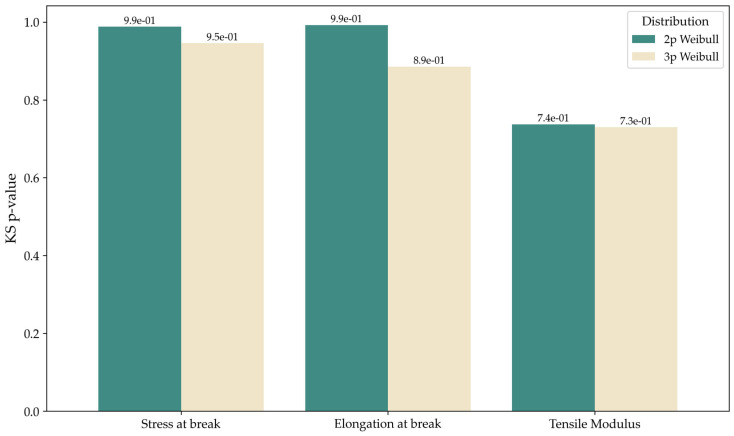
Weibull model evaluation for rCF before carding.

The tensile properties of recycled carbon fibers are experimentally identified, as described in Section 2.2, and the data are fitted to two Weibull models, the two-parameter model and the three-parameter model. The parameters estimated by MLE are then compared with the actual distribution using the K-S test. Figure 4 gives an example of the K-S test *p*-values calculated on recycled fibers before carding, for the three tensile parameters under study, and for the Weibull distributions with two and three parameters.

For fibers before carding, the highest K-S test *p*-value for tensile modulus, stress at break, and elongation at break corresponds to the two-parameter Weibull model. The *p*-values, which reflect the fit between the theoretical and experimental models, measured for the two-parameter Weibull model, are higher than for the three-parameter model for rCF before carding. The fitting between theoretical and experimental values gave a *p*-value for stress at break and elongation at break of 0.988 and 0.992, respectively, for the two-parameter distribution, which suggests a high degree of fit with the experimental model, with the value being close to 1. For the tensile modulus, the value of the *p*-value is lower, at 0.737, but still slightly higher than the one obtained with the three-parameter model for fibers before carding, also suggesting a reasonable fit.

Table A1 (in Appendix A) gives the different values computed with Weibull with two and three parameters for the different rCF/PP blend ratios. Using the K-S test value, the K-S test *p*-value, and SSE calculations to choose the best model, it was decided that the two-parameter Weibull distribution be used.

Having these values, for generalization purposes and to avoid overfitting for each of the cases, the two-parameter Weibull (Equation (1), with $\sigma_{u}=0$) model was used throughout this study. Additionally, it is worth noting that the choice of the two-parameter Weibull model offers a balance between model complexity and goodness of fit, enhancing the interpretability and robustness of the analysis.

### 3.2. Tensile Properties

**Figure 5:**
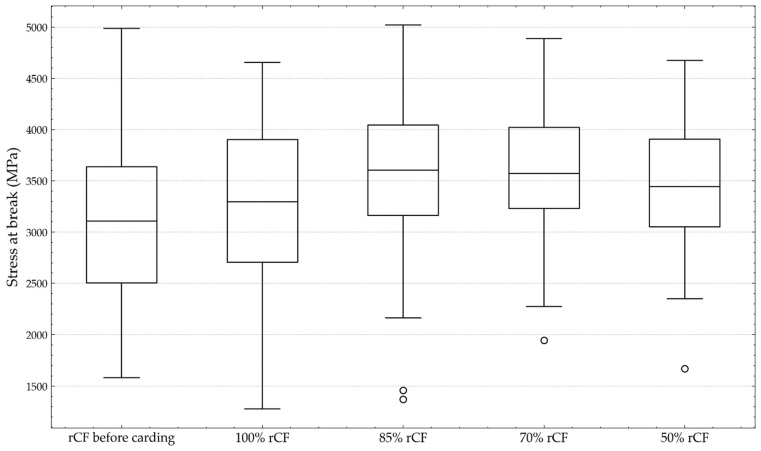
Stress-at-break boxplots for different blending ratios.

Before each tensile strength test, the fiber diameter is measured using an OLYMPUS DSX1000 optical microscope. The diameters of the recycled carbon fibers measured for each batch (Table 1) showed similar variability to virgin carbon fibers, with CV% ranging from 3.37 to 6.11% [30]. After carding, the average diameter of the fibers decreases very slightly, while the standard deviation increases. However, all values remain within the standard deviations of one another, indicating a limited impact of the carding process on fiber diameter. Additionally, it was observed that the transverse section shape of the fibers was not modified by the carding process and remains circular. Figure 5 provides further insights into the variability of fiber properties depending on the fiber ratio. The dispersion observed in these tests highlights significant variability, supported by the standard deviations reported in Table 1.

The coefficient of variation (CV%) for the tensile modulus is approximately 11%, while for stress and strain at break, it reaches 23%. This level of dispersion is typical for single-fiber tensile tests and underscores the necessity of employing statistical models, particularly probabilistic approaches, to characterize the behavior of these fibers at failure. The variability shown in Table 1 for tensile modulus, stress, and elongation at break highlights the need to use probabilistic models to better analyze and understand the dispersion in these mechanical properties. For the 50–50% ratio, a sample size of 25 tests was retained following post-processing of the tensile data. This number was confirmed to be sufficient through complementary statistical analyses. Levene’s test for homogeneity of variance [37] (*p* = 0.7144) indicated no significant differences in variance across all batches, and a cumulative mean analysis demonstrated that 25 tests were sufficient to achieve result stability, ensuring the sample size reliably represented the fiber properties.

#### 3.2.1. Stress at Break

From the values in Table 1, the average stress at break for each batch of fiber showed no significant change in terms of standard deviations. Before carding, the average stress at break of the rCF was 3143 ± 773 MPa. All the stress-at-break values after carding, regardless of the proportion of polypropylene fiber in the blend, are within this standard deviation value.

**Figure 6:**
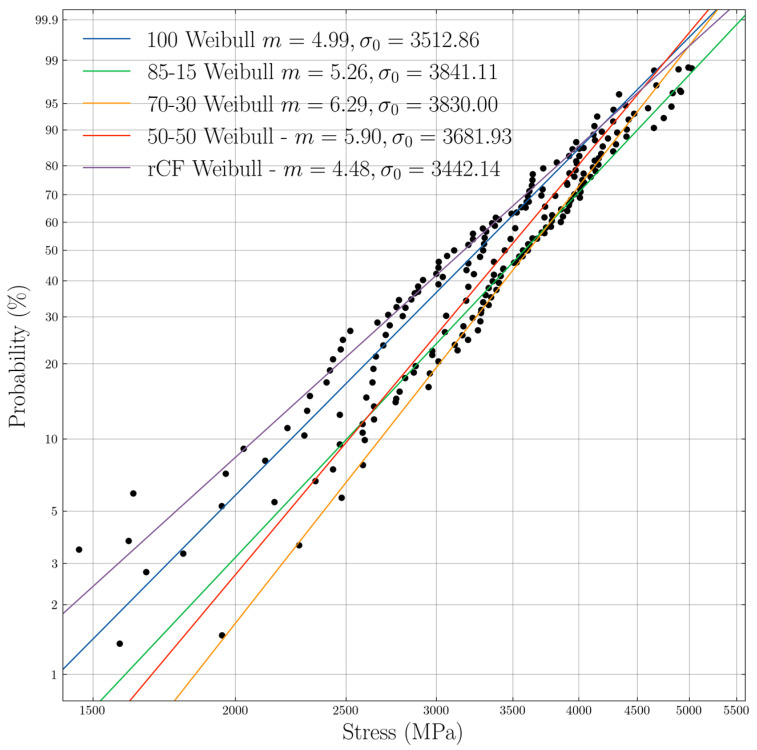
Weibull two-parameter probability plot—stress at break.

In order to determine the differences in distributions of the stress at break according to the PP fiber ratio, these values are also studied using a Weibull modulus from the two-parameter model in Figure 6.

The MLE technique is used to obtain a mean value and a standard deviation on the calculated Weibull modulus values. It can already be noted that the number of tests has a significant impact on the standard deviation of the Weibull modulus. In fact, for the 50–50 blend, whose dataset contains 20 fewer usable trials than the 100% rCF batch, the standard deviation rises from 0.59 to 0.92 (Table 2).

The *p*-values provided in Table 2, particularly those close to 1, indicate a good fit of the experimental data to the Weibull model, apart from the 100% rCF batch where the *p*-value is slightly lower at 0.89, suggesting a slightly less consistent fitting for this set.

The Weibull modulus values increase with the carding process (100% rCF) compared with fibers recycled before carding (Figure 6), indicating a reduced spread in stress at break data. In relation to the average values and Weibull moduli for each of the fibers extracted from the webs, it can be observed that on the criterion of stress at break, carding has no significantly damaging impact on the carbon fibers.

The slight increase in ($m,\;\sigma_{0}$) can be attributed to the selection of fibers with a length longer than 30 mm for the test. Carding eliminates the weakest fibers, which are broken during the process. This reduces the probability of these weak fibers being collected during the tensile test. Otherwise, weak fibers present in the initial batch of fibers prior to carding might be selected and included in the tensile test, leading to lower measured strength values.

Adding the crimped PP fiber leads to decreased data scattering regarding the stress at break with a higher Weibull’s scale parameter ($\sigma_{0}$). This effect varies with the PP ratio. The highest impact is noted around 30% for a PP–fiber ratio (from 4.99 to 6.29). This leads to larger increases in the scale parameter in comparison with raw rCF before carding, which can be attributed to advantage gained in regards to the elimination of weak fibers, which drafts the distribution to a higher scale parameter. Further, it can be supposed that the crimped PP fiber (with higher inter-fiber entanglement) induces a higher pull-out force exerted to individualize a fiber from a fiber tuft.

#### 3.2.2. Tensile Modulus

To avoid variations in stress due to the resolution of the force sensor, the tensile modulus is calculated by linear regression in the deformation range between 0.1 and 0.6%, in accordance with standard NF ISO 11566. The average tensile modulus of recycled fibers calculated before carding is equal to 186.2 ± 20.8 GPa (Table 1) which places them at the lower end of the range compared with standard modulus (SM) virgin carbon fiber [24].

After carding (Table 1) the tensile modulus of rCFs extracted from webs increases, first comparatively to the tensile modulus of rCF before carding, also and in relation to the ratio of PP in webs. This modulus reaches 206.4 GPa for the fibers extracted from the webs with a ratio of 50–50.

**Figure 7:**
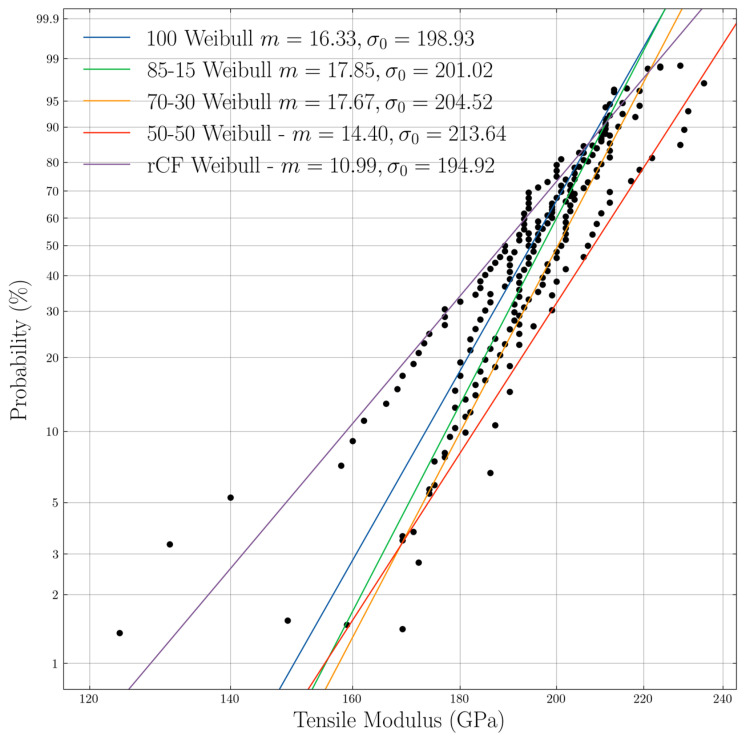
Weibull two-parameter probability plot—tensile modulus.

In parallel with the increase in the tensile modulus values for carded fibers compared to fibers before carding, a corresponding increase in the Weibull modulus on the tensile modulus value is observed (Figure 7 and Table 3). For the 50–50 ratio, with the lower *p*-value, the higher standard deviation on the Weibull modulus can be explained with the lower number of experimental data points.

**Figure 8:**
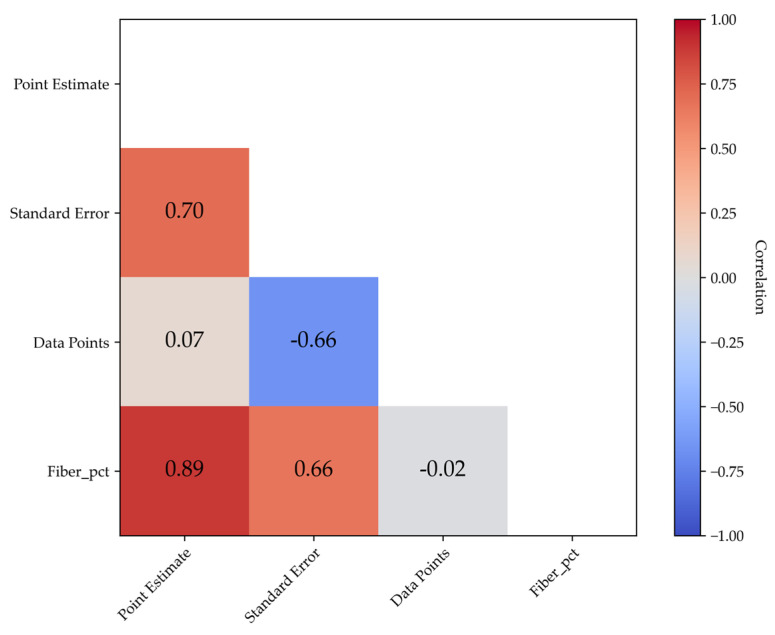
Heatmap for Weibull modulus on the tensile modulus.

The heatmap shown in Figure 8 can be used to describe the relationships between two variables by calculating the Pearson coefficient. An absolute value close to one indicates a strong linear correlation between two variables. The “Fiber_pct” variable corresponding to the fiber content in the web shows a strong and positive correlation (0.89) with the value of the Weibull modulus in the tensile modulus, denoted “Point Estimate” in Figure 8.

This indicates a homogenization of the tensile modulus values with carding, on the one hand, but also with the increase in the PP ratio in the web where the tested fibers are extracted. The Weibull modulus values reach a peak for blends 70–30% and 85–15%, suggesting an optimum value of PP ratio in the webs produced.

**Figure 9:**
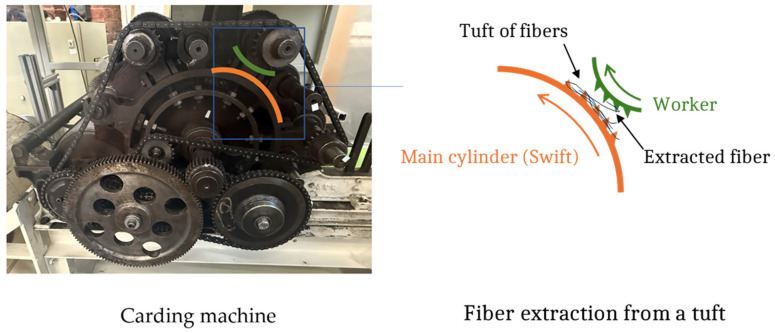
Fiber pull-out from tuft during carding.

This increase in tensile modulus values is highlighted by scientific studies describing the phenomenon of the stiffening of the carbon fibers either during the tensile test with the increase in deformation [38] or during cyclic tensile tests where a slight increase in the tensile modulus of the fibers was observed due to a realignment of the graphite crystal microstructure along the fiber axis [39]. In the fiber extraction process using the carding machine, mechanisms of fiber rupture can occur. When a carding tooth catches a fiber loop, as shown in Figure 9, if the fiber is trapped at both ends within the fiber tuft, it breaks.

Also, if the force applied to extract the fiber exceeds its resistance, the fiber will also break. The extracted fiber is subjected to successive tensile forces, which can either cause it to break or to be extracted from a tuft of fibers. Linked to the observation made by Bunsell et al. [39] on cyclic tensile tests for carbon fibers, this mechanism within the carding machine provides the beginnings of an explanation for the increase in tensile modulus values and stiffening, but also for the homogenization of values resulting in an increase in Weibull modulus values.

#### 3.2.3. Elongation at Break

To complete this investigation, a study of elongation at break values was also carried out. Before carding, the average elongation at break value of the rCF was 1.57 ± 0.37% (Table 1).

Since carbon fiber is a rigid fiber, the trends shown for stress at break also apply to elongation at break. The average values of strain at break of carded rCF, regardless of the level of PP fiber in the blend, remain stable and within the standard deviation of fibers recycled before carding (Table 1).

**Figure 10:**
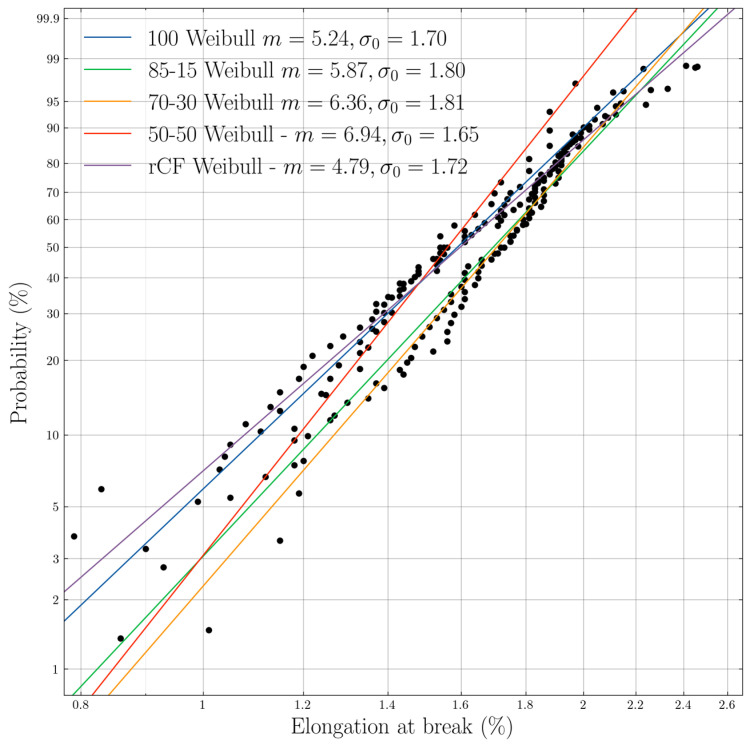
Weibull two-parameter probability plot—elongation at break.

The Weibull modulus values (Table 4 and Figure 10) show the same trend as that seen for the stress-at-break values. A slight increase in Weibull modulus values was observed with carding and the addition of PP fiber to the blend. For instance, the modulus increased from 4.79 for non-carded fibers to 6.94 for carded carbon fibers in a 50–50 mix, reflecting a homogenization of values as a result of carding and the elimination of weak fibers.

The *p*-values in Table 4. further confirm the good fit of the Weibull two-parameter model to the experimental data, except for the 15–85 rCF blend, where the *p*-value is noticeably lower. This deviation suggests that the two-parameter Weibull model may not fully capture the distribution of elongation-at-break data for this specific blend. While no definitive explanation is available for this anomaly, it is worth noting that when a three-parameter Weibull model is applied (Table A1), the *p*-value for the 15–85 rCF blend remains similarly low.

## 4. Discussion

The influence of the proportion of thermoplastic polypropylene (PP) fibers on the mechanical properties of recycled carded carbon fibers (rCF) was investigated using a statistical Weibull approach. The comparison between two-parameter and three-parameter Weibull models highlights the better fitting of the two-parameter Weibull model for describing the tensile properties of rCF before carding, leading to its use for the study. Four different web compositions were prepared with varying rCF/PP blend ratios (100/0%, 85/15%, 70/30%, and 50/50%). The experimental results lead to the following conclusions:

- Impact of the proportion of PP fibers on mechanical properties:
  The addition of crimped PP fibers influences the mechanical properties of rCF. Regarding stress at break, the presence of PP fibers decreases the disparity among fibers and shifts the stress distribution to higher values. This improvement is attributed to the removal of the weakest fibers during the blending process. For the tensile modulus, the inclusion of PP fibers further amplifies the slight increases observed after carding alone, particularly in blends with 85/15% and 70/30% rCF/PP ratios. The addition of PP fibers ensures a more consistent distribution of tensile modulus values, as reflected by the increased Weibull modulus. In contrast, elongation at break values remain stable across all PP blend ratios, with minor improvements in uniformity, as shown by slight increases in the Weibull modulus.
- Impact of the carding process on mechanical properties:
  Carding improves the mechanical consistency of rCF, particularly in webs composed of 100% rCF. The process leads to a slight increase in tensile modulus values for fibers extracted from these webs, indicating improved alignment and structural uniformity. Carding increased the Weilbull modulus values of stress at break, tensile modulus, and elongation at break across all rCF/PP blends. Moreover, carding facilitates the intimate integration of PP fibers with rCF, creating hybrid blends.

Overall, the study demonstrates the potential of carding rCF in conjunction with PP to produce carded webs. A major innovation of this work lies in the integration of a mechanical and statistical approach to evaluate the effects of carding and PP fiber blending. The application of the Weibull statistical model provided insights into the mechanical behavior of rCF. However, the limitations of the Weibull model in exploring mechanical variations have been acknowledged. An upcoming publication will investigate alternative statistical models, including normal, lognormal, and three-parameter Weibull distributions.

Future research will also focus on studying the impact of varying carding parameters on fiber quality and mechanical performance.

In conclusion, the incorporation of thermoplastic fibers in the production of hybrid composites leads to modifications in the mechanical properties of recycled carbon fibers. This impact can be effectively analyzed through a combination of processing techniques and statistical analysis, as demonstrated in this study. Furthermore, the proposed approach can be extended to other carding parameters and textile processing techniques, providing deeper insights into the mechanical properties and ensuring greater consistency in the processing of recycled carbon fibers.

## Figures and Tables

**Table 1:** Fibre properties before and after carding according to their ratio in the webs.

	Data Points	Diameter (µm)		Stress at Break (MPa)		Tensile Modulus (GPa)		Elong. at Break (%)	
	Numb.	Mean	Std.	Mean	Std.	Mean	Std.	Mean	Std.
100% rCF	45	7.36	0.45	3221	769	192.8	13.7	1.57	0.36
15–85% rCF	49	7.21	0.46	3406	741	195.5	12.1	1.57	0.29
30–70% rCF	47	7.18	0.34	3563	656	198.5	13.6	1.68	0.30
50–50% rCF	25	7.10	0.48	3413	685	206.4	15.9	1.53	0.27
rCF bef. Card.	51	7.41	0.25	3143	773	186.2	20.8	1.57	0.37

**Table 2:** Two-parameter Weibull modulus values of stress at break for each fiber ratio.

	Data Points	Two-Parameter Weibull Modulus		Kol. Smirn.
		Mean	Std.	*p*-Value
100% rCF	45	4.99	0.59	0.89
15–85% rCF	49	5.26	0.59	0.99
30–70% rCF	47	6.29	0.71	0.99
50–50% rCF	25	5.90	0.92	0.99
rCF bef. carding	51	4.48	0.47	0.99

**Table 3:** Two-parameter Weibull modulus values of tensile modulus for each fiber ratio.

	Data Points	Two-Parameter Weibull Modulus		Kol. Smirn.
		Mean	Std.	*p*-Value
100% rCF	45	16.33	1.84	0.83
15–85% rCF	49	17.85	1.89	0.82
30–70% rCF	47	17.67	1.99	0.84
50–50% rCF	25	14.40	2.18	0.76
rCF bef. carding	51	10.99	1.18	0.74

**Table 4:** Two-parameter Weibull modulus values of elongation at break for each fiber ratio.

	Data Points	Two-Parameter Weibull Modulus		Kol. Smirn.
		Mean	Std.	*p*-Value
100% rCF	45	5.24	0.62	0.99
15–85% rCF	49	5.87	0.64	0.43
30–70% rCF	47	6.36	0.69	0.96
50–50% rCF	25	6.94	1.10	0.96
rCF bef. carding	51	4.79	0.52	0.99

## Data Availability

The original contributions presented in the study are included in the article, further inquiries can be directed to the corresponding author.

## Appendix A

**Table A1:** K-S test and SSE parameter values for 2 and 3-parameter Weibull model for each rCF/PP ratio.

	Two-Parameter Weibull									Three-Parameter Weibull								
rCF Ratio	Tensile Modulus			Stress at Break			Elongation at Break			Tensile Modulus			Stress at Break			Elongation at Break		
	SSE	KS	*p*-Val	SSE	KS	*p*-Val	SSE	KS	*p*-Val	SSE	KS	*p*-Val	SSE	KS	*p*-Val	SSE	KS	*p*-Val
50–50	2.31 × 10^−4^	1.28 × 10^−1^	7.56 × 10^−1^	5.23 × 10^−9^	6.76 × 10^−2^	9.99 × 10^−1^	9.45 × 10^−1^	9.64 × 10^−2^	9.57 × 10^−1^	1.40 × 10^−4^	8.56 × 10^−2^	9.85 × 10^−1^	5.06 × 10^−9^	6.80 × 10^−2^	9.99 × 10^−1^	9.11 × 10^−1^	9.67 × 10^−2^	9.56 × 10^−1^
30–70	7.90 × 10^−5^	8.64 × 10^−2^	8.44 × 10^−1^	3.18 × 10^−8^	5.38 × 10^−2^	9.98 × 10^−1^	4.85 × 10^−1^	7.12 × 10^−2^	9.57 × 10^−1^	8.68 × 10^−5^	7.75 × 10^−2^	9.19 × 10^−1^	3.18 × 10^−8^	6.09 × 10^−2^	9.90 × 10^−1^	5.32 × 10^−1^	6.91 × 10^−1^	9.67 × 10^−1^
85–15	4.22 × 10^−4^	8.69 × 10^−2^	8.22 × 10^−1^	2.42 × 10^−8^	5.71 × 10^−2^	9.94 × 10^−1^	1.49 × 10^0^	1.22 × 10^−1^	4.27 × 10^−1^	3.48 × 10^−4^	8.28 × 10^−2^	8.62 × 10^−1^	4.21 × 10^−4^	8.69 × 10^−2^	8.22 × 10^−1^	1.48 × 10^0^	1.16 × 10^−1^	4.80 × 10^−1^
100	2.43 × 10^−7^	9.01 × 10^−1^	8.26 × 10^−1^	3.63 × 10^−8^	8.32 × 10^−2^	8.88 × 10^−1^	9.79 × 10^−2^	6.36 × 10^−2^	9.87 × 10^−1^	1.94 × 10^−4^	7.87 × 10^−2^	9.22 × 10^−1^	3.65 × 10^−8^	8.19 × 10^−2^	8.98 × 10^−1^	1.03 × 10^−1^	7.03 × 10^−2^	9.68 × 10^−1^
rCf b. c.	4.63 × 10^−5^	9.27 × 10^−2^	7.37 × 10^−1^	3.90 × 10^−8^	5.97 × 10^−2^	9.88 × 10^−1^	1.30 × 10^−1^	5.74 × 10^−2^	9.92 × 10^−1^	4.61 × 10^−5^	9.28 × 10^−2^	7.29 × 10^−1^	4.43 × 10^−8^	7.05 × 10^−2^	9.46 × 10^−1^	2.25 × 10^−1^	7.87 × 10^−1^	8.86 × 10^−1^
